# Neuroendocrine and Peptidergic Regulation of Stress-Induced REM Sleep Rebound

**DOI:** 10.3389/fendo.2016.00163

**Published:** 2016-12-23

**Authors:** Ricardo Borges Machado, Deborah Suchecki

**Affiliations:** ^1^Department of Psychology, Psychosomatic Research Group, Universidade Ibirapuera, São Paulo, Brazil; ^2^Department of Pharmacy, Psychosomatic Research Group, Universidade Ibirapuera, São Paulo, Brazil; ^3^Department of Psychobiology, Escola Paulista de Medicina, Universidade Federal de São Paulo, São Paulo, Brazil

**Keywords:** sleep, stress, prolactin, CLIP, serotonin, CRH, homeostasis, REM sleep

## Abstract

Sleep homeostasis depends on the length and quality (occurrence of stressful events, for instance) of the preceding waking time. Forced wakefulness (sleep deprivation or sleep restriction) is one of the main tools used for the understanding of mechanisms that play a role in homeostatic processes involved in sleep regulation and their interrelations. Interestingly, forced wakefulness for periods longer than 24 h activates stress response systems, whereas stressful events impact on sleep pattern. Hypothalamic peptides (corticotropin-releasing hormone, prolactin, and the CLIP/ACTH_18–39_) play an important role in the expression of stress-induced sleep effects, essentially by modulating rapid eye movement sleep, which has been claimed to affect the organism resilience to the deleterious effects of stress. Some of the mechanisms involved in the generation and regulation of sleep and the main peptides/hypothalamic hormones involved in these responses will be discussed in this review.

## Introduction

The purpose of the present review is not to present a detailed description of the neural basis of sleep generation and maintenance. For that, we refer to a number of previous review papers that cover this subject (1–5).

**Figure 1:**
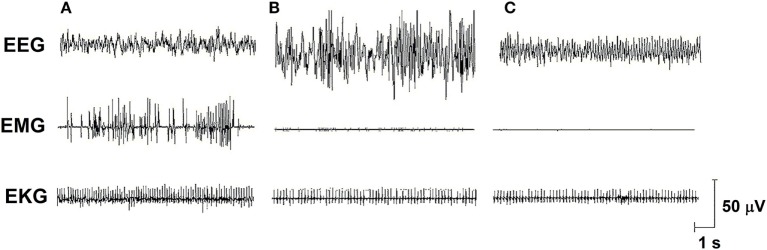
**Electro-oscillographic signs of wakefulness and sleep in the *Wistar* rat**. **(A)** Active wake (with low voltage and fast EEG frequency, concomitant a high EMG activity and EKG is fast), **(B)** non-rapid eye movement sleep (NREMS—high amplitude and slow wave EEG, activity in EMG is low and EKG is low), **(C)** rapid eye movement sleep (REMS—theta 6–8 Hz activity is present in this medial EEG, EMG is almost quiet, and EKG shows an intermediate activity). EEG, electroencephalogram (obtained from a medial frontoparietal bipolar deviation); EMG, electromyogram (from the trapezius muscle); EKG, electrocardiogram (from intercostal electrodes). Signs were calibrated with 50-µV pulse. Data from our group.

Sleep is a fundamental behavior for the individual’s survival and is redundantly regulated by the interaction of several neurotransmitter and neuropeptide systems acting on several brain structures, mainly located in the hypothalamus and brain stem [for review, see Ref. (6)]. In humans and rodents, sleep is classified into two main stages: non-rapid eye movement sleep (NREMS) and rapid eye movement sleep (REMS), which electroencephalographic features, in rats, are represented in Figure 1. In humans, NREMS encompasses three stages, N1, N2, and N3, characterized by synchronized, high amplitude, and low frequency cortical waves, whereas in rats, NREMS or slow wave sleep can be distinguished into two substages, differing in the amplitude of these slow waves (low- and high-amplitude waves). REMS or paradoxical sleep (used for rats, since they show very little eye movements) is characterized by desynchronized, high frequency, low-amplitude cortical waves, very similar to wakefulness, and hippocampal theta waves. In addition, muscle atonia is a main tonic feature of this sleep stage (7, 8).

## Sleep Regulation and Homeostasis

### Circadian and Homeostatic Mechanisms

Sleep is regulated by a combination of homeostatic and circadian mechanisms. The homeostatic process refers to sleep needs or pressure, and the circadian one, entrainment to the light/dark cycle. Besides the homeostatic factor, the circadian aspect is important and is related to the animal’s expression of daily preference for sleep/rest. Also, the duration of the sleep episodes appears to be greater in animals that are at the top of the food chain, since preys need to monitor the environment constantly to ensure their integrity, thus sleeping very short bouts (9, 10). The interaction between homeostatic (called “S process”) and circadian factors (called “C process”) in sleep regulation led some authors to propose a model in which the two processes would act together. Sleep begins wherever there is a conjunction of larger homeostatic pressure (need for sleep) and greater circadian predisposition (proximity to the phase of the cycle that sleep normally occurs), whereas it ends when this interaction decreases (11, 12).

Sleep homeostasis depends, among many factors, on the length and quality of the preceding waking period. Therefore, longer periods of waking lead to greater compensatory sleep, also known as rebound sleep. Interestingly, brief periods of sleep deprivation (SD) (3–6 h) result only in increased NREMS without affecting REMS (13). A total of 12–24 h of total sleep deprivation increases both NREMS and REMS (14–16), whereas total SD for 96 h induces a very pronounced increase in REMS (17). In 1960, few years after the discovery of REMS, William Dement (18) reported, for the first time in humans, that selective REMS deprivation induces a compensatory increase of this specific phase, e.g., REMS rebound. These effects have been replicated in rats, using the platform method that produces a complete suppression of REMS and some loss of NREMS (19, 20).

### Effects of Stress

It is interesting to note that REMS deprivation induces the activation of the hypothalamic–pituitary–adrenal (HPA) axis, with increased production (21, 22) and release (23) of corticotropin-releasing hormone (CRH), and increased ACTH (24, 25) and corticosterone plasma levels (22, 26–28). This constellation of neuroendocrine changes indicates the stressful nature of this manipulation, thereby leading some authors to question whether sleep rebound would be an outcome of stress exposure. It soon became clear that stressful events can alter sleep pattern in a stimulus- and length-related fashion, inasmuch as immobilization stress increases REMS (29), social conflict (30) and exposure to cold (31) induce NREMS, whereas unpredictable footshock stress decreases REM sleep (32, 33). One to two hours of immobilization stress in the beginning of the dark phase, increase REMS, but longer periods of stress blunt the expression of sleep rebound (34), similar to what is seen with repeated immobilization (29). These opposite effects between acute and chronic exposure to the stress seem to be due to an adaptation phenomenon, which is also observed with footshock stress (32, 35). Either increased or reduced stress-induced REMS appear to be related to corticosterone secretion, in an inverted U shape fashion (36, 37).

## Hormonal Regulation of Stress-Induced Sleep Rebound

### HPA Axis

Abnormal functioning of the HPA axis substantially changes the sleep pattern, as observed in Addison’s disease patients who exhibit adrenal insufficiency and display more NREMS and less REMS (34); this sleep abnormality can be corrected by corticoid replacement therapy (38). Conversely, patients with Cushing’s syndrome, who exhibit exaggerated cortisol secretion, display less NREMS and more awakenings during the night, which can also be corrected by interventions that decrease production of glucocorticoids (GCs) (39, 40). These findings strongly suggest that optimal GC concentrations are essential for normal sleep patterns in humans (41, 42). In rats, a similar phenomenon is also observed, with high levels of corticosterone being especially detrimental to NREMS (43).

The 41-aminoacid peptide CRH, its mRNA, and receptors are increased in stress situations, such as electric footshock (44), immobilization (45), restriction of food (46), and sleep deprivation (21, 23). CRH stimulates the release of other proopiomelonocortin (POMC)-derived peptides, including β-endorphin and alpha-melanocyte-stimulating hormone (α-MSH) (47–49). It differentially stimulates the activity of the prohormone convertases (PC1 and PC2, mainly), which cleave POMC in its bioactive peptides (50, 51).

CRH reduces REM and NREMS and increases awakenings when injected intravenously (52) or after i.c.v. administration (53), whereas CRH type 1 receptor (CRH-R1) antagonists promote sleep (54, 55). CRH effects on sleep homeostatic regulation are also evident, for its administration immediately after sleep deprivation increases REMS rebound (56). Conversely, α-helical-CRH_9−41_, a CRH-R1 antagonist inhibits REMS rebound induced by immobilization (57). Recently, we have demonstrated that both i.c.v. CRH or α-helical-CRH_9−41_ administration during REMS deprivation impairs sleep homeostasis, thereby decreasing the length of REMS episodes in the theta band energy (6.0–9.0 Hz), and decreases the time of REM and NREMS in the recovery period (58).

### Corticotropin-Like Intermediate Peptide (CLIP or ACTH_18–39_)

**Figure 2:**
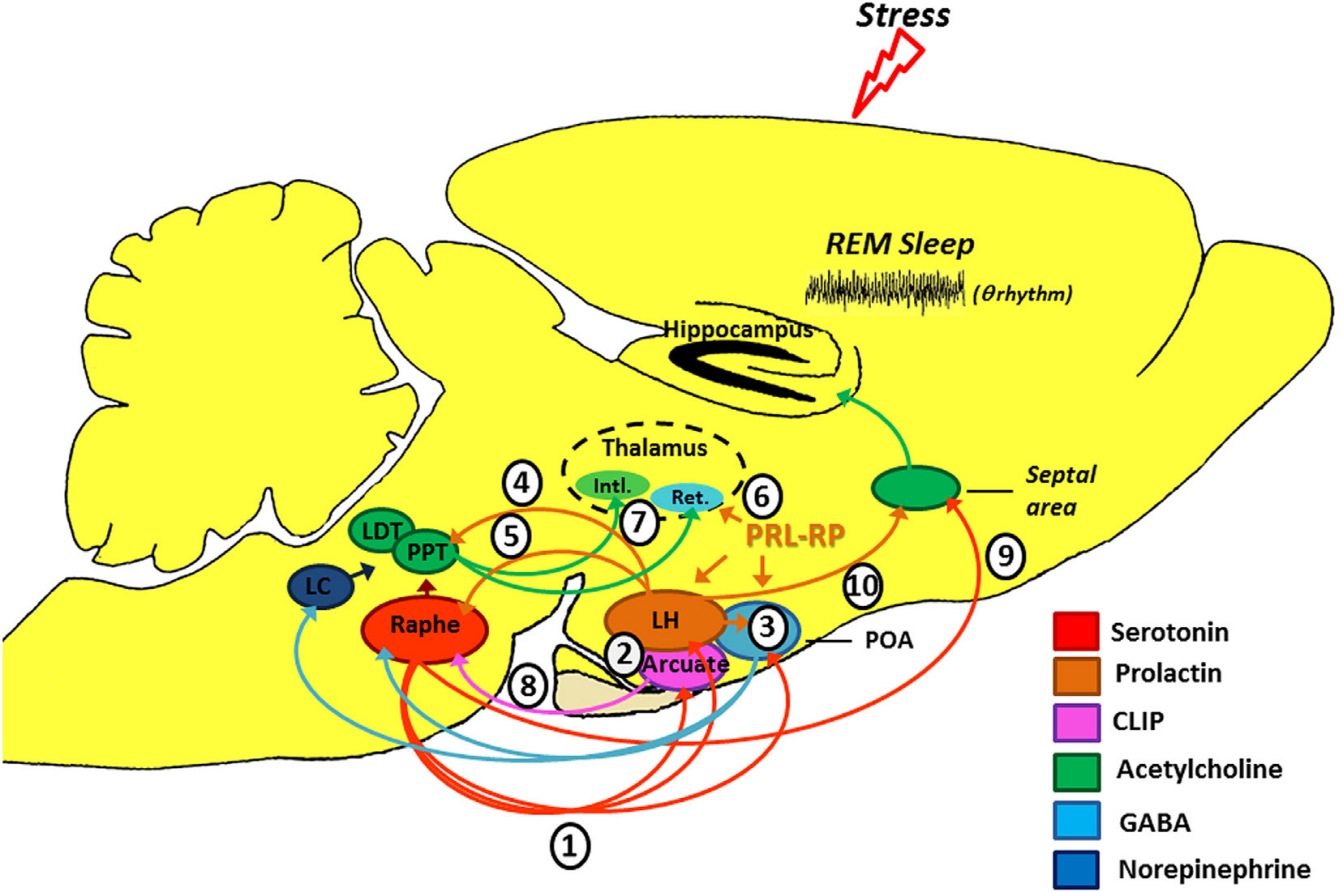
**Schematic model of the possible modulation of stress-induced rapid eye movement sleep rebound by PRL, CLIP, and PRL-RP**. LC, *locus coeruleus*; LDT, laterodorsal tegmental nucleus; PPT, pedunculopontine tegmental nucleus; LH, lateral hypothalamic area; POA, preoptic area; Arcuate, arcuate nucleus of hypothalamus; Intl., thalamic intralaminar nuclei; Ret., reticular thalamic nucleus. For more details, please refer to the main text.

CLIP is a POMC derivative, well known for inducing long REMS episodes (59, 60). ACTH cleavage to CLIP and α-MSH is mediated by prohormone convertase 2 (PC2) and is stimulated by serotonin, *via* 5-HT_2C_ receptors (61–63). This process occurs in the melanocytes of the pituitary *pars intermedia* and in two other distinct locations in the brain: the arcuate nucleus—Arc (and peri-arcuate) in the basomedial hypothalamus and in a cell group of the nucleus of the solitary tract (NST) (64, 65). There are also CLIP-containing fibers, originating in the Arc and projecting to the lateral, paraventricular, basal and preoptic hypothalamic regions, dorsal and medial raphe nucleus (DRN and MRN, respectively), and septal area (66–70) (Figure 2).

Initial studies on the effects of stress-induced REMS rebound show that 1 h of immobilization stress increases REMS 3 h after the end of the stressor, a time point that corresponds to the increased content of phosphorylated CLIP in the Arc (71, 72). Conversely, micro-infusion of CLIP into the DRN increases REMS, similar to that observed after acute immobilization stress (73–75). It has been hypothesized that stress induces 5-HT release from the raphe nuclei, which projections to the Arc lead to CLIP processing from POMC. When the stress finishes, 5-HT is reuptaken, whereas CLIP synthesis and release from the Arc still go on. Interestingly, immunoreactive CLIP fibers are found in the DRN, which are postulated to trigger the dendritic release of serotonin (76) by a process of extracellular diffusion (67). The dendritic release of 5-HT within the DRN inhibits further 5-HT synthesis and release, *via* 5HT_1A_ autoreceptors (77, 78), a process commonly observed during REMS (79, 80). Confirmation of this mechanism was obtained by electrical stimulation of the DRN leading to release of 5-HT and indol compounds in the Arc, followed, 3 h later, by increased REMS (81) (Figure 2). It is worth mentioning that REMS induced by CLIP, more precisely the amino-terminal fragment ACTH_20–24_ (Val-Lys-Tyr-Pro), is characterized by extremely long episodes, usually above 7 min. This effect on REMS is not obtained with ACTH (ACTH_1–39_) or other active fragments, such as ACTH_18–24_ (60).

### Prolactin and Prolactin-Releasing Peptide

Prolactin (PRL) is a polypeptide hormone consisting of 199 amino acid residues (in rats and mice, by 197 amino acid residues), which is synthesized and secreted by the anterior pituitary lactotrophs, under tonic dopaminergic inhibitory control (82, 83); the main function of pituitary PRL is to stimulate lactation in mammals (84). In humans, PRL is secreted mostly in the second half of the night (85), with a very clear circadian rhythm (86).

This hormone exhibits anxiolytic effects (87), inhibits the development of stress-induced gastric ulcers (88), and mitigates hormonal and neuronal responses to various stressors (89, 90). PRL also has an important neurotrophic effect and is involved with neurogenesis in various brain structures and cell cultures (91–96), preventing stress-induced decrease in neurogenesis in an animal model of chronic stress (96). PRL is closely associated with physiological stress response (97–99), and immunoreactive neurons are found in the lateral hypothalamus, Arc, and surrounding areas, thereby innervating other hypothalamic nuclei, amygdala, nucleus accumbens, olfactory cortex, septum, the reticular formation region, parabrachial region, locus coeruleus, periaqueductal gray, and DRN (100), and presenting practically the same distribution of POMC and its derivatives, as evidenced by double labeling studies (101). Interestingly, such structures are particularly involved in the regulation of sleep and stress response (Figure 2).

Numerous evidence indicates that PRL may be another mediator of stress-induced REMS rebound phenomenon. On the one hand, movement restriction (102) and ether vapor exposure (103) increase PRL plasma levels and time in REMS in rodents. On the other hand, PRL infusion in the dorsolateral hypothalamus or in the lateral ventricle increases REMS during the light phase (104, 105). Recently, we also showed that footshock stress applied concomitantly to REMS deprivation produces substantial REMS rebound, by virtue of very long REM episodes. Plasma PRL and hypothalamic serotonin (5-HT) were the likely mediators of the major increase in REMS episode length (37). Serotonin stimulates PRL release (106, 107) and PRL also increases serotonin synthesis (108–111) (Figure 2). In addition, both PRL micro-infusion into the DRN (112) and systemic administration of PRL increases REMS (113), whereas anti-PRL antibody suppresses REMS in rats (114), and this phase of sleep is naturally reduced in mice genetically deficient for PRL (115).

Prolactin-releasing peptide (PRL-RP) acts on a seven-transmembrane G protein-coupled receptor promoting PRL release *in vitro* and *in vivo* (116, 117). Central PRL-RP administration increases REMS in rats in parallel to PRL circulating levels (118, 119). Intriguingly, high PRL-RP concentrations also increase NREMS and have no effect on REMS (118, 119). In a somewhat contradictory way, PRL-RP has the ability to reduce the oscillatory activity in sections of the reticular thalamic tissue (120), which plays a fundamental role in the generation of NREMS (121).

## Concluding Remarks

Several experimental stressors (1) (122, 123) and SD protocols (124, 125) promote serotonin release from the dorsal raphe (see Figure 2). Ascending serotonergic projections stimulate CLIP and PRL production and release by the Arc and lateral hypothalamic area (LH), respectively (2), which depend on gene transcription and protein synthesis (hence, the rebound happens only a few hours after exposure to the stressful stimulus). Serotonin- and PRL-dependent self-stimulating neural circuits (and probably PrRP of the anterior preoptic area) (3), producing GABAergic inactivation of the DRN and LC on pontine nuclei (pedunculopontine tegmental/laterodorsal tegmental) (126). Furthermore, PRL can also activate the pontine cholinergic neurons directly (4) (127). Prolactinergic projections to DRN (5) (100, 101) induce serotonin release (112) that, at first, can feedback on the system leading to additional production and release of CLIP and PRL. However, 5-HT excess can stimulate self-receptors (5-HT_1A_) in the DRN, thereby inhibiting its activity (128, 129). All these phenomena contribute positively to REM sleep expression. Additionally, PrRP reduces the oscillatory activity of the reticular thalamic neurons (Ret.) (6) (120), an important structure for the generation of synchronized sleep (121), which also has positive effect on REM sleep. Without the inhibitory influence of the DRN and LC (130, 131), pontine cholinergic nuclei (7) stimulate the thalamic intralaminar nuclei (Intl.), which induces cortical activation during desynchronized sleep and inhibits GABAergic neurons of thalamic reticular nucleus (132–134). We should emphasize that CLIP has inhibitory action (8) on serotonin release by the DRN (71), thereby contributing also to halt the suppressive activity that it has on pontine nuclei. It is noteworthy that there is a serotonergic ascending pathway, from the DRN to the septal area (9) that is responsible for the inhibitory activity in the cholinergic basal forebrain area, essential for hippocampal theta rhythm during REM sleep (135). This region also has a high density of PRL receptors (136) and can also be a potential site of prolactin action. Thus, PRL (10) also contributes to REM sleep expression.

REMS in mammals is involved with various functions, such as brain maturation of neonates (10), maintenance of minimal brain activity during sleep [which would allow a quick wake up and avoid a possible coma during sleep (137)], memory consolidation (138, 139), and maintenance of brain monoaminergic neurotransmitter systems (140–143), among others. Recently, however, studies have brought the attention to the importance of REMS in emotion regulation and the main adaptive function of REMS rebound after stress. This is well exemplified by studies showing that patients with posttraumatic stress disorder (PTSD) display longer latency to REMS, and short REM episodes and sleep fragmentation (144, 145). In a recent work, Mellman and colleagues assessed the sleep of individuals after a traumatic event and found a negative correlation between the duration of REMS episodes and development of PTSD. Furthermore, PTSD patients display reduced EEG high frequency activity during REMS, indicating low cognitive activation during this sleep phase (146), thereby pointing to the need for long and consolidated REMS events to elaborate and integrate traumatic memories in conscious level (147). Therefore, we do not hesitate to propose, for reasons of evolutionary logic, that in rodents, increased REMS observed after some kinds of stress has a similar role (148).

## Author Contributions

RM and DS contributed equally to this work.

## Conflict of Interest Statement

The authors declare that the research was conducted in the absence of any commercial or financial relationships that could be construed as a potential conflict of interest.
